# Pharmacological manipulation of liver fibrosis progression using novel HDAC6 inhibitors

**DOI:** 10.1111/febs.70062

**Published:** 2025-03-14

**Authors:** Maria Teresa Borrello, Dusan Ruzic, Hannah Paish, Eleanor Graham, Amy L. Collins, Rebecca Scott, Sam Higginbotham, Branko Radovic, Glyn Nelson, David Bulmer, Lee A. Borthwick, Stuart M. Robinson, Jeremy French, John Moir, Steve A. White, Colin Wilson, Sanjay Pandanaboyana, John Hammond, Rohan Thakkar, Wasfi Alrawashdeh, Rodrigo Figueiredo, Milos Petkovic, Katarina Nikolic, Fiona Oakley, Derek A. Mann, Jelena Mann

**Affiliations:** ^1^ Newcastle Fibrosis Research Group, Bioscience Institute, Faculty of Medical Sciences Newcastle University UK; ^2^ School of Pharmacy and Pharmaceutics, Faculty of Health Sciences and Wellbeing University of Sunderland UK; ^3^ Department of Pharmaceutical Chemistry, Faculty of Pharmacy University of Belgrade Serbia; ^4^ Bioimaging Unit, Faculty of Medical Sciences Newcastle University UK; ^5^ FibroFind Newcastle upon Tyne UK; ^6^ Department of Hepatobiliary Surgery Newcastle upon Tyne Hospitals NHS Foundation Trust UK; ^7^ Department of Organic Chemistry, Faculty of Pharmacy University of Belgrade Serbia; ^8^ Newcastle University Centre for Cancer Newcastle University UK

**Keywords:** HDAC6 inhibitors, histone deacetylase inhibitors, human precision cut liver slices, liver fibrosis, therapy

## Abstract

Chronic liver injury characterized by unresolved hepatitis leads to fibrosis, potentially progressing to cirrhosis and hepatocellular carcinoma. Effective treatments for halting or reversing liver fibrosis are currently lacking. This study investigates the potential of HDAC6 as a therapeutic target in liver fibrosis. We synthesized two selective HDAC6 inhibitors, DR‐3 and FDR2, and assessed their effects on hepatic stellate cell (HSC) activation and liver fibrosis using human precision cut liver slices (hPCLS). Molecular docking, deacetylation inhibition assays, and various cellular assays were employed to evaluate the specificity and anti‐fibrotic efficacy of these inhibitors. DR‐3 and FDR2 demonstrated high selectivity for HDAC6 over HDAC1, significantly inhibiting HSC activation markers and fibrogenic gene expression. Both inhibitors increased acetylation of α‐tubulin and suppressed TGF‐β1‐induced SMAD signaling in HSCs. In human precision cut liver slices (hPCLS), DR‐3 and FDR2 reduced fibrogenic protein levels and collagen deposition. The selective inhibition of HDAC6 by DR‐3 and FDR2 effectively reduces HSC activation and fibrogenesis in liver models, supporting further investigation of HDAC6 inhibitors as potential anti‐fibrotic therapies.

## Graphical abstract

Chronic liver injury can lead to severe conditions such as cirrhosis and liver cancer. Unfortunately, there are no effective treatments to halt or reverse liver fibrosis, which makes new therapeutic strategies essential. This study focuses on the role of histone deacetylases (HDACs) in liver fibrosis, particularly HDAC6. We synthesized two new small molecule inhibitors, DR‐3 and FDR2, which specifically target HDAC6. These inhibitors were shown to reduce fibrosis in both human liver cells and tissue models. The findings suggest that HDAC6 inhibitors could be promising candidates for treating liver fibrosis and possibly other fibrotic diseases. Figure created using Biorender.

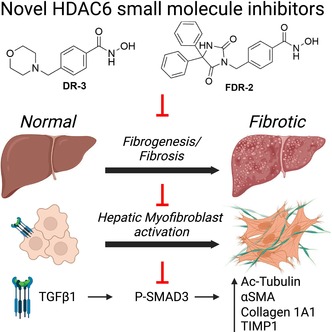

**Abbreviations**
aHSC: activated hepatic stellate cells
Alk5i: TGF‐β receptor/ALK5 inhibitor
CD1: catalytic domain 1
CD2: catalytic domain 2
CLD: chronic liver disease
ECM: extracellular matrix
HDAC: histone deacetylase
HDAC6smi's: HDAC6 small molecule inhibitors
HDACi: histone deacetylase inhibitors
hPCLS: human precision cut liver slices
HSC: hepatic stellate cells
IC50: half maximal inhibitory concentration
LDH: lactate dehydrogenase
MAPK/ERK: mitogen‐activated protein kinase/extracellular signal‐regulated kinase
MASH: metabolic associated steatohepatitis
PDGF: platelet‐derived growth factor
PI3K/AKT: phosphoinositide 3‐kinase/protein kinase B
p‐SMAD3: phosphorylated SMAD3
PSR: Picrosirius red
qHSC: quiescent hepatic stellate cells
TGF‐β1: transforming growth factor beta 1
TIMP‐1: tissue inhibitor of metalloproteinase‐1
TRAIL: TNF‐related apoptosis‐inducing ligand
YAP/TAZ: yes‐associated protein/transcriptional coactivator with PDZ‐binding motif
α‐SMA: alpha‐smooth muscle actin

## Introduction

Unresolved liver injury is characterized by chronic hepatitis, which stimulates a progressive fibrogenic process that, if unchecked, can advance to cirrhosis and/or hepatocellular carcinoma. At present, there are no effective clinically approved treatments for halting or reversing liver fibrosis other than to suppress the underlying cause of liver damage. As liver disease now accounts for 4% of all early deaths worldwide, there is a pressing need for novel therapeutic approaches [1].

Central to the fibrogenic process is the local production of wound‐healing activated myofibroblasts, which promote the net deposition of fibril‐forming extracellular matrix (ECM) proteins through ECM protein synthesis and the secretion of tissue inhibitor of metalloproteinase‐1 (TIMP‐1) which acts to prevent ECM protein degradation [2, 3, 4]. Myofibroblasts are also a rich source of soluble proinflammatory factors, and as such, contribute to the persistence of inflammation [5, 6]. Additionally, these cells are highly proliferative, migratory, and are able to promote the spread of fibrosis from the initial site of tissue damage [7].

The major source of myofibroblasts in the damaged liver is the resident quiescent hepatic stellate cells (qHSC) which are a population of non‐parenchymal cells that are zonally distributed in both portal‐ and central‐vein locations. HSC exhibit remarkable plasticity, and in response to liver injury and/or inflammation, transdifferentiate (or “activate”) into proliferative pro‐fibrogenic myofibroblasts [4, 8]. Resolution of injury is accompanied by a decline in the numbers of activated hepatic stellate cells (aHSC) which is achieved either through apoptosis or “inactivation” and reversion to a quiescent state [7, 9]. However, chronic injury stimulates continued HSC activation, which is regulated by a combination of paracrine and autocrine signals, of which transforming growth factor beta 1 (TGF‐β1) and platelet‐derived growth factor (PDGF) are important for promoting ECM deposition and myofibroblast proliferation, respectively [10, 11].

We and others have previously highlighted the pivotal role played by epigenetic regulators in the control of HSC activation and their potential as targets for the design of anti‐fibrotic molecules [12, 13, 14]. Histone deacetylases (HDACs) are a family of 18 protein deacetylases that are classified into distinct classes: class I (HDAC 1, 2, 3 and 8), class IIa (HDAC 4, 5, 7 and 9), class IIb (HDAC 6 and 10) and class IV (HDAC 11) are Zinc‐dependent enzymes, whereas the class III HDACs (Sirtuins 1–7) are NAD‐dependent enzymes [15]. HDACs have been reported to be involved in the regulation of fibrosis initiation and progression in multiple organs, and small molecule inhibitors of HDACs (HDACi) have been shown to have potent anti‐fibrotic activities in animal models of tissue damage [16, 17, 18]. The majority of HDACs are upregulated in models of HSC activation and liver fibrosis, which complicates selecting a specific HDAC for targeting in fibrosis. However, HDAC2, HDAC6, and HDAC8 are notable for their downregulation during the resolution of liver fibrosis [19], which may indicate a role for these deacetylases in the progression of fibrosis.

HDAC6 is predominantly cytoplasmic and interacts with a variety of non‐histone protein substrates involved in biological processes relevant to HSC and fibrosis, including cell migration, proliferation, and apoptosis [20]. A diverse number of signaling molecules of relevance to inflammation and fibrosis are controlled by HDAC6, including the PI3K/AKT and MAPK/ERK pathways [21, 22]. Moreover, studies in the human lung epithelial‐like cell line A549 showed that HDAC6 is required for TGFβ1‐induced epithelial–mesenchymal transition involving control of Smad3 signaling [23]. Collectively, these observations suggest HDAC6 may be a rational target in fibrosis, as does the fact that *Hdac6* ^*−/−*^ mice display no obvious developmental or health‐related defects [24].

In this report, we describe the synthesis of two selective HDAC6 small‐molecule inhibitors (DR‐3 and FDR2) and demonstrate their ability to suppress HSC activation and inhibit fibrosis induced by a combination of TGFβ1 and PDGF in human precision‐cut liver slices (hPCLS).

## Results

### Synthesis of novel small molecule inhibitors selective for HDAC6

**Fig. 1:**
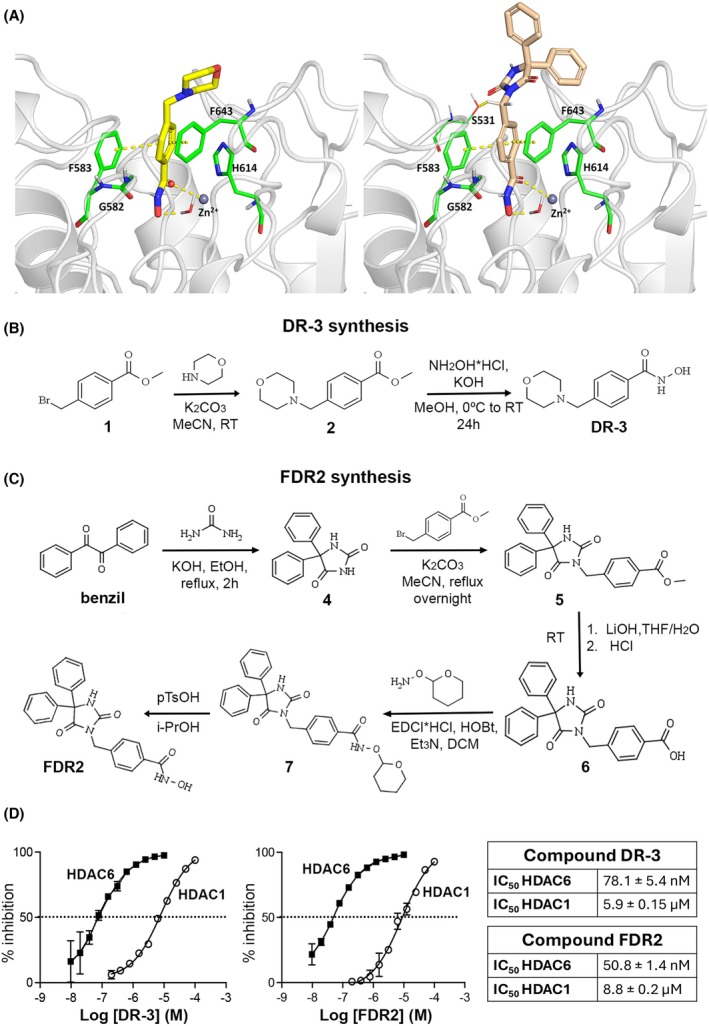
Synthesis and enzymatic evaluation of novel small molecule inhibitors selective for HDAC6. (A) Structure‐based models of HDAC6 inhibitors prepared using the PyMOL Molecular Graphics System version 3.0 (Schrodinger, LLC), highlighting the molecular framework and binding interactions. (B, C) Schematic representation of the synthetic pathways for compounds DR‐3 and FDR2, illustrating the chemical reactions and intermediate steps involved in their synthesis. (D) Dose–response curves for DR‐3 and FDR2, showing their comparative inhibition of HDAC6 versus HDAC1 activity. The assays were performed in parallel using purified enzymes, with the results indicating the percentage of histone substrate deacetylation inhibition, and data are mean ± SEM of *n* = 3 repeats.

The HDAC6 small molecule inhibitors (HDAC6smi's) DR‐3 and FDR2 are derivatives of phenylhydroxamic acid, a potent but non‐selective HDAC6 inhibitor [25]. To design selective HDAC6 inhibitors, we targeted a key structural feature of HDAC6, namely, the wider catalytic pocket of HDAC6 compared to class I HDACs [26]. The morpholine (for DR‐3) and 5,5‐diphenyl imidazolidine 2,4‐dione (for FDR2) CAP groups were initially introduced onto the structure of phenylhydroxamic acid to exploit the wider catalytic pocket of HDAC6 (Fig. 1A) and generate selective HDAC6 inhibitors. The chemical synthesis of DR‐3 and FDR2 is outlined in the schematic illustrated in Fig. 1B,C, and the detailed synthetic steps are supplied in the Materials and methods section and Figs S1–S5. The original X‐ray crystal structure of HDAC6 (PDB: 5G0J) revealed two independent catalytic domains, CD1 and CD2, lined by an interdomain [27]. CD2 exhibits broader substrate specificity than CD1, and crystal structures of CD2‐inhibitor complexes have revealed atomic details of the active site that enable structure‐based design of novel inhibitors. Our synthetic approach was based on evidence that aromatic linkers contribute to improved selectivity toward HDAC6, as does the attachment of rigid and/or bulky CAP groups to the phenylhydroxamate moiety of the hydroxamic‐based molecule [28]. Figure 1A shows the predicted binding modes for DR‐3 and FDR2, with their phenyl linkers anchored within the aromatic crevice formed by the F583 and F643 residues of the CD2 active site [29, 30]. The carbonyl oxygen in position 2 of the imidazolidine capping group in FDR2 accepts the hydrogen bond from the side chain S531, which presents a unique interaction pattern observed for the CD2 catalytic pocket. Compared to FDR2, the capping group of compound DR‐3 (morpholine ring) did not establish significant interactions with the side chain residues located at the outer rim of HDAC6. This observation additionally confirms the importance of the aromatic linker in the structure of selective HDAC6 inhibitors compared to the nature of the capping group. Molecular docking analysis revealed that the hydroxamate portion of both DR‐3 and FDR2 establishes bidentate hydroxamate‐Zn^2+^ coordination geometries with the catalytic Zn^2+^ ion. Conclusively, analysis of selective binding modes of DR‐3 and FDR2 is in alignment with their *in vitro* HDAC selectivity profiles (Fig. 1D, IC_50_ values on enzymes).

To determine the selectivity of the molecules for HDAC6, we carried out a deacetylation inhibition assay for purified HDAC1 and HDAC6 enzymes using the HDAC‐Glo I/II assay kit (Promega). IC_50_ values demonstrated selectivity in the nm range for both compounds (78.1 ± 5.4 nm and 50.8 ± 1.4 nm for DR‐3 and FDR2 respectively) compared with μm values for inhibition of HDAC1, indicating a 100‐fold selectivity of the two inhibitors for HDAC6 compared with HDAC1 (Fig. 1D).

### Selective HDAC6smi suppresses fibrogenic features of human LX‐2 hepatic myofibroblasts

**Fig. 2:**
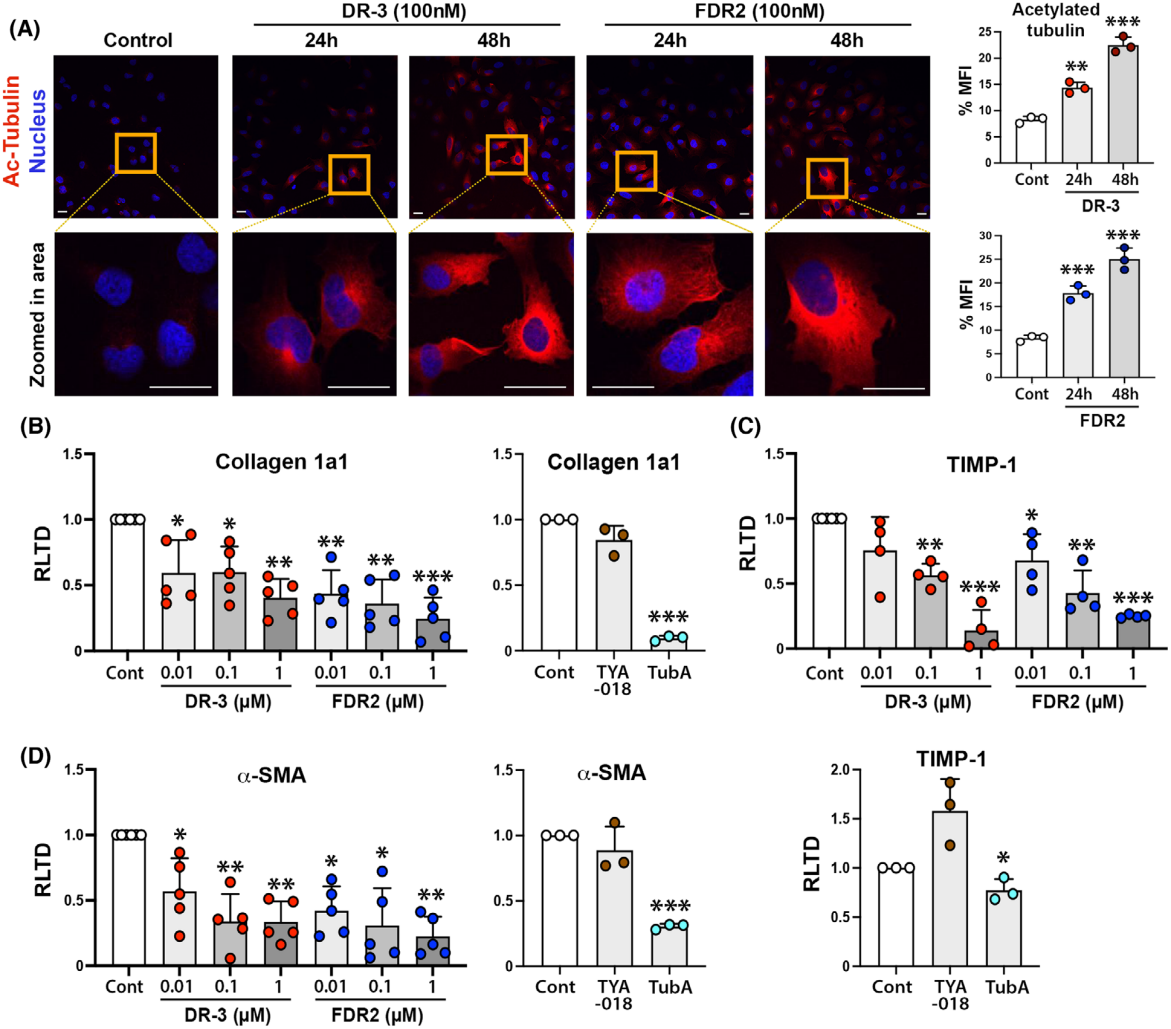
Selective HDAC6smi suppresses fibrogenic features of the human hepatic stellate cell line LX‐2. (A) Representative images of acetylated tubulin‐stained LX‐2 cells after 24 and 48 h of treatment with HDAC6 small molecule inhibitors DR‐3 and FDR2 (100 nm); Quantification of acetylated tubulin signal using imagej, capturing Mean Fluorescence Intensity (MFI) expressed as a percentage compared to vehicle control, *n* = 3 (as notified by three dots on the graphs); (B–D) mRNA levels of Collagen 1a1, TIMP‐1, and αSMA expressed as relative level of transcription difference (RLTD) in LX‐2 cells at the indicated concentration of HDAC6smi's DR‐3 and FDR2, 3 μm TYA‐018 and 50 μm Tubastatin A after 24 h of treatment. Scale bar = 20 microns. Data are mean ± SEM, from two individual experiments performed in either duplicate or triplicate, each datapoint represents a different experimental sample. Significance was calculated using a one‐way ANOVA with a Tukey's *post hoc* test, and *P < 0.05, **P < 0.01, or ***P < 0.001.

The HDAC6smi's were first tested for their ability to inhibit HDAC6 *in vitro* using the human LX‐2 hepatic myofibroblast line [31]. Tubulins (α and β) are cytoskeletal proteins with functions that are under the regulation of acetylation modifications. Reversible acetylation of α‐tubulin is enabled by HDAC6‐mediated global deacetylation of the protein at microtubules where HDAC6 localizes with the microtubule motor complex [32]. Of relevance to fibrosis, TGF‐β1 induces acetylation of α‐tubulin and is required for downstream YAP/TAZ signaling in myofibroblasts, particularly under conditions of low matrix stiffness [33, 34]. Exposure of LX‐2 to 100 nm DR‐3 and FDR2 for 24 h resulted in increased levels of acetylated α‐tubulin as determined by immunofluorescence microscopy (Fig. 2A). Exposure at 48 h was associated with a further modest increase in the post‐translational modification. We next determined effects of the HDACsmi's on the expression of transcripts for Collagen 1a1, TIMP‐1, and α‐SMA which in combination provide a robust determination of the fibrogenic activity of myofibroblasts. Following a 24 h treatment, both compounds were able to suppress the expression of these three transcripts, although FDR2 appeared more effective than DR‐3 in these assays (Fig. 2B–D). In parallel, we tested the HDAC6 inhibitors, TYA‐018 and Tubastatin A, using doses published for human cardiac fibroblasts [35] and murine chondrocytes [36] respectively. In LX‐2 cells, 24 h or 3 μm TYA‐018 did not change fibrogenic gene expression, whereas 50 μm Tubastatin A suppressed the expression of Collagen 1a1, TIMP‐1, and α‐SMA to levels comparable to those achieved using 1 μm DR‐3 and FDR2 (Fig. 2B–D). In summary, DR‐3 and FDR2 are effective for relieving HDAC6‐mediated deacetylation of α‐tubulin and exert anti‐fibrogenic effects at the transcript level in the human LX‐2 hepatic myofibroblast line.

### Selective HDAC6smi suppresses the fibrogenic characteristics of primary rat HSC

**Fig. 3:**
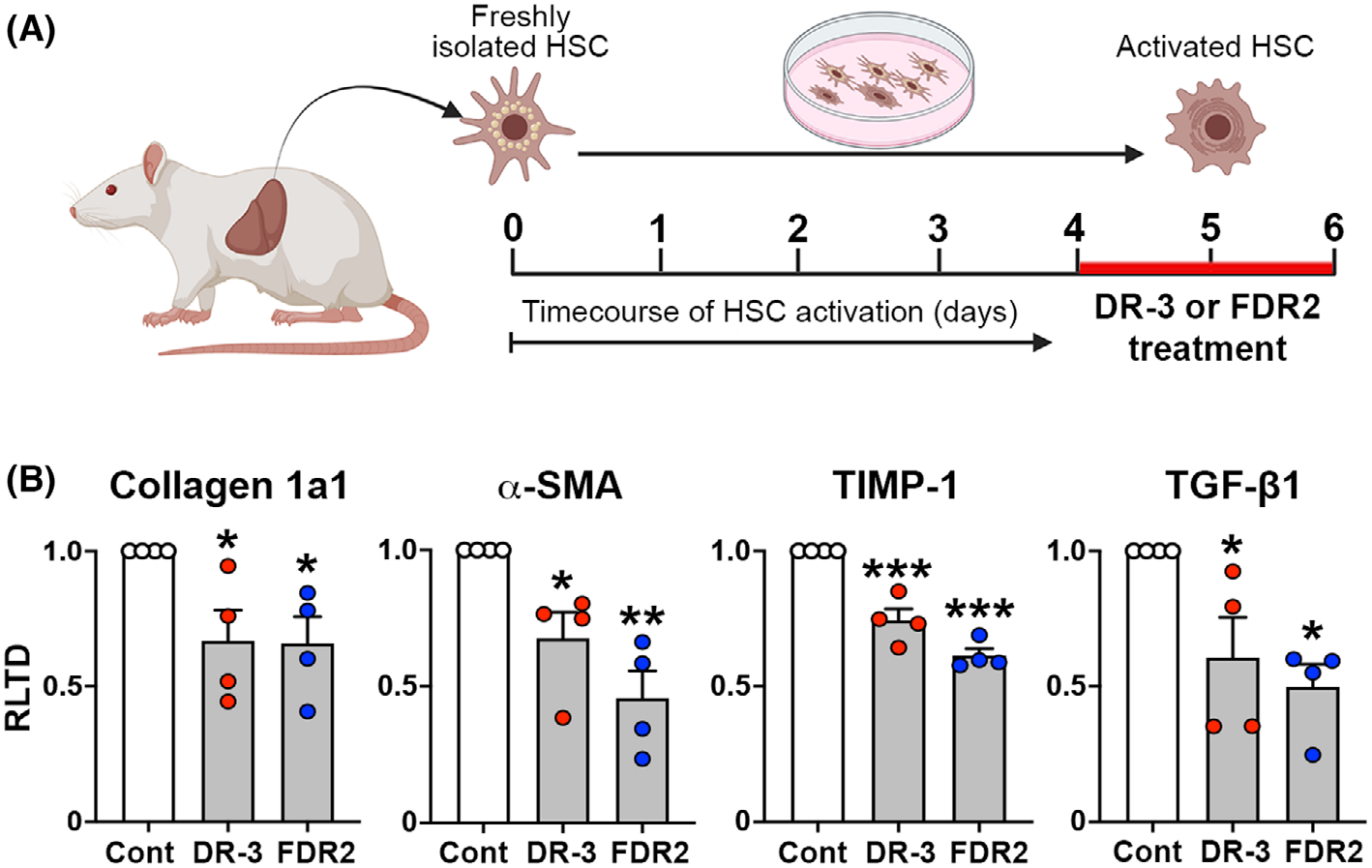
Selective HDAC6smi's suppress fibrogenic characteristics of primary rat HSC. (A) Schematic illustrating the isolation, culture and treatment of rat hepatic stellate cells (rHSC) with HDAC6 small molecule inhibitors DR‐3 or FDR2. (B) mRNA levels of fibrogenic gene transcripts expressed as relative level of transcription difference (RLTD) of primary rat HSC cells cultured for 4 days after isolation and treated with 100 nm of DR‐3 or FDR2 for 24 h. *P < 0.05, **P < 0.01, ***P < 0.001.

To corroborate the antifibrogenic properties of DR‐3 and FDR2 observed with LX‐2 cells, we carried out similar experiments using primary rat HSC. As illustrated in the schematic of Fig. 3A, freshly isolated HSC were prepared and subsequently cultured in full‐serum supplemented media on plastic for 4 days to adopt an activated phenotype [37, 38]. Cultures were then exposed to 100 nm DR‐3 and FDR2 for 24 h prior to harvesting, isolation of RNA, and quantification of fibrogenic gene transcripts. Both HDAC6smi's suppressed the expression of Collagen 1a1, α‐SMA, and TIMP‐1 transcripts (Fig. 3B). In addition, 100 nm DR‐3 and 100 nm FDR2 treatment suppressed HSC expression of TGF‐β1 mRNA, indicating the potential of the inhibitors to interfere with the autocrine control of the fibrogenic activities of activated HSC.

### HDAC6smi suppresses TGF‐β1‐induced profibrogenic SMAD signaling

**Fig. 4:**
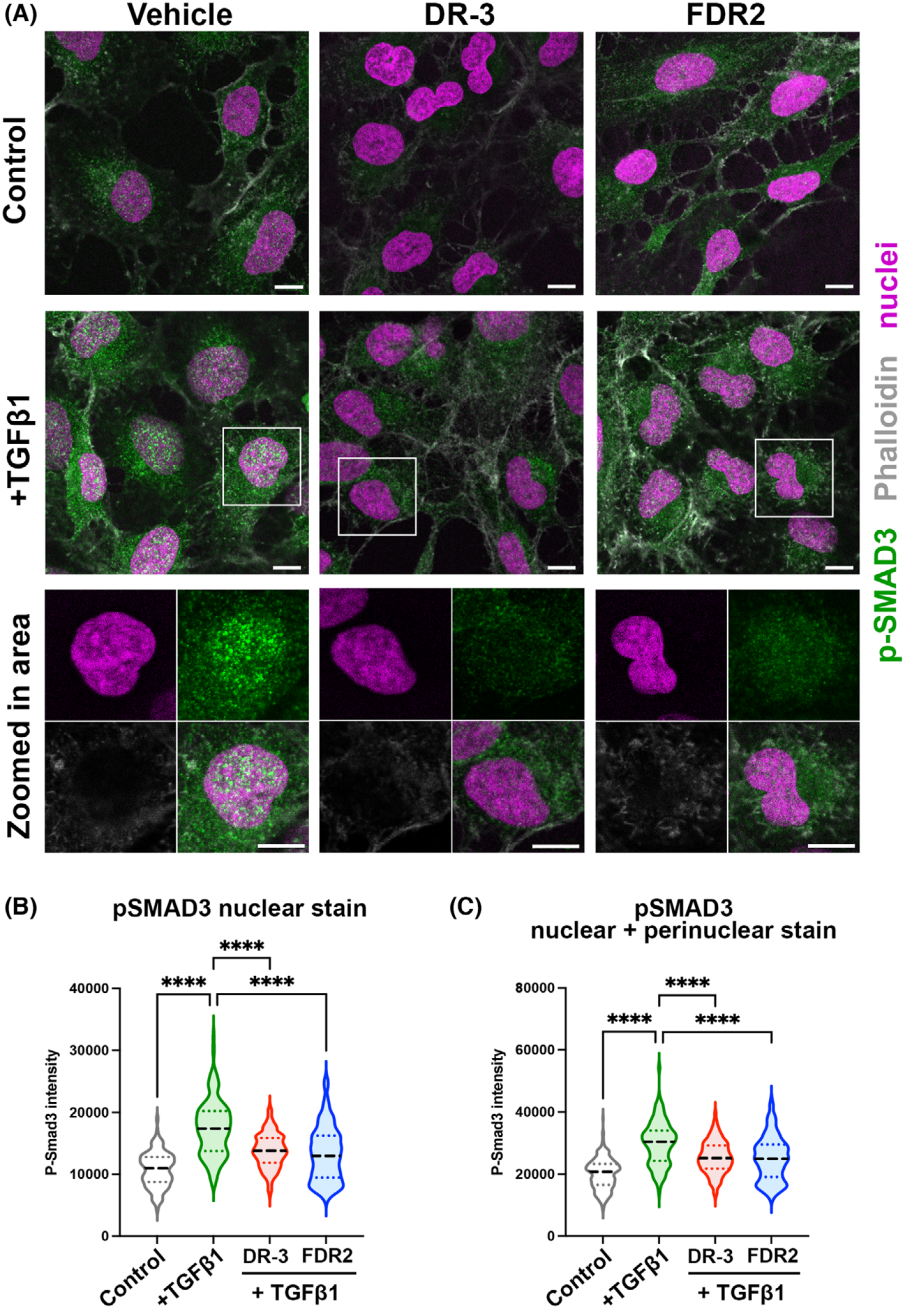
HDAC6smi suppresses TGFβ1 induced profibrogenic SMAD signaling. (A) Immunofluorescence imaging of LX2‐cells following overnight treatment with HDAC6 inhibitor DR‐3 (100 nm) and stimulation with TGFβ1 (5 ng·mL^−1^) for 30 min. The stimulation shows the appearance of punctate nuclear staining for pSMAD3, indicative of the anticipated activation for vehicle control and reduced nuclear pSMAD3 upon HDAC6 small molecule inhibitor treatment. (B, C) Quantification of nuclear (B) or nuclear and perinuclear (C) pSMAD3 stain in control or 15 min TGFβ1 treatment ± DR‐3 or FDR2. Scale bar = 10 microns. Data shown as median values (dashed line), with the upper and lower quartiles (dotted lines), and represent a minimum of 135 cells/group. Significance was calculated using a one‐way ANOVA with a Tukey's *post hoc* test, and ****P < 0.0001.

Following the engagement of TGF‐β1 with TGF‐β receptors on HSC, downstream phosphorylation of the signaling proteins SMAD2 and SMAD3 results in their interaction with SMAD4 and subsequent nuclear translocation of the complex to enable activation of canonical TGF‐β1‐dependent gene transcription [39, 40]. The DNA binding activity of SMAD3 is critical for the induction of the fibrogenic activities of HSC, whereas, by contrast, SMAD2 lacks DNA binding activity and appears to be predominantly antifibrogenic through its ability to stimulate TRAIL‐dependent apoptosis of HSC [41, 42]. As HDAC6 has been implicated in the control of TGF‐β1 signaling, we were interested to ask if the HDAC6smi's have an influence on TGF‐β1‐induced nuclear translocation of SMAD3. Immunofluorescence imaging of LX‐2 cells confirmed low levels of detectable cytoplasmic phosphorylated SMAD3 (p‐SMAD3) and nuclear pSMAD3 protein prior to treatment with TGF‐β1 (Fig. 4A–C). Following TGF‐β1 treatment, there was a noticeable increase in pSMAD3 in the cytoplasm and appearance of punctate nuclear staining for pSMAD3 indicative of the anticipated translocation (left hand panels in Fig. 4A). Levels of TGF‐β1‐induced cytoplasmic p‐SMAD3 were significantly reduced in cells exposed to DR‐3 and FDR2 (middle and right‐hand panels in Fig. 4A,C) compared to controls treated with TGF‐β1 alone (left hand panel, Fig. 4A). Higher magnification enabled detection of a few discrete punctate p‐SMAD3‐containing nuclear complexes in both DR‐3 and FDR2 treated cells, but when quantified, they were significantly less abundant than were detected in cells treated with TGF‐β1 alone (Fig. 4B).

### HDAC6smi's have antifibrogenic activities in human precision‐cut liver slice cultures

**Fig. 5:**
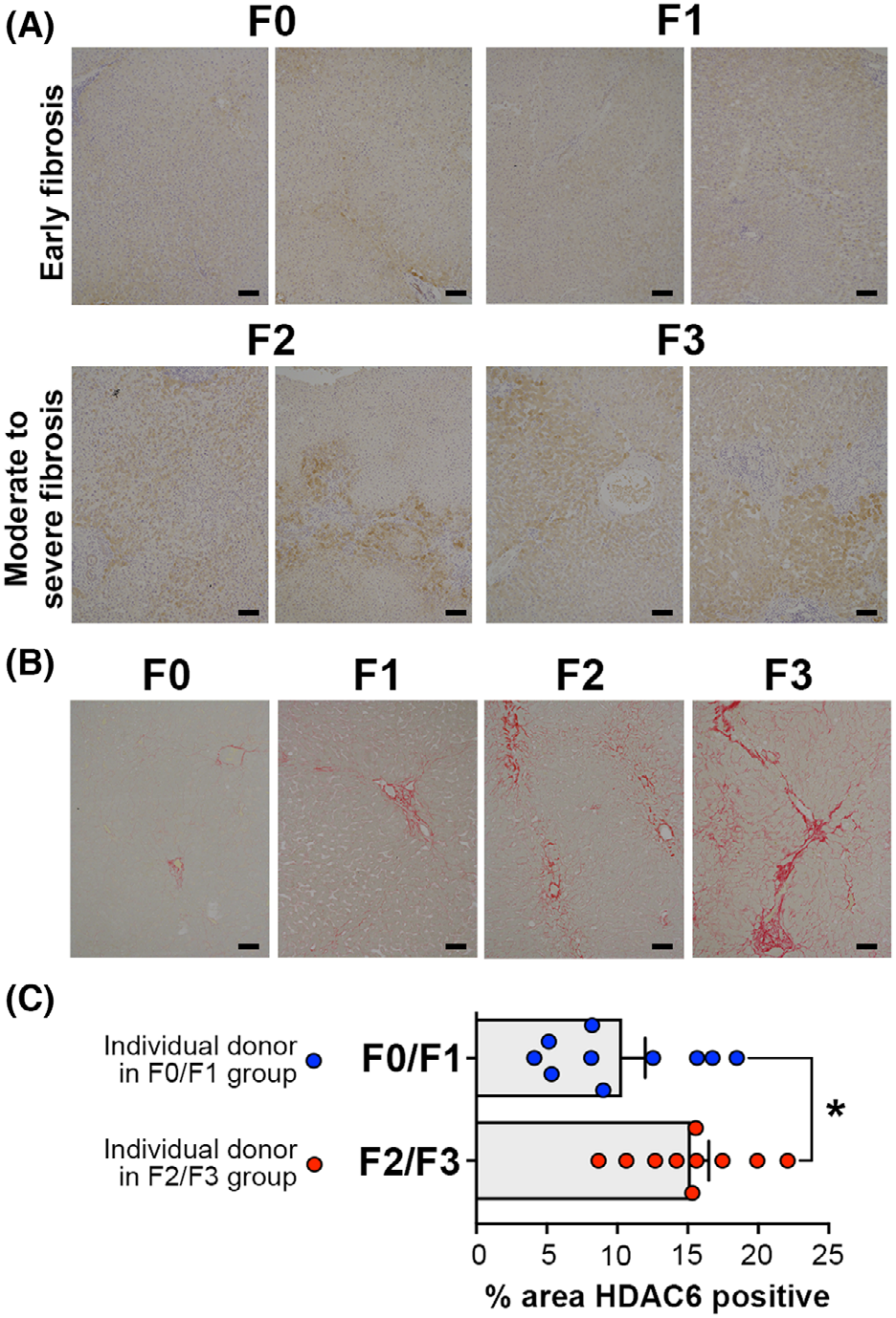
HDAC6 expression in human livers proportionally increases with fibrotic conditions. (A) Immunohistochemistry of human liver sections from donors ranging from early to advanced fibrosis, categorized as F0–F1 (normal or mild fibrosis) and F2–F3 (significant to advanced fibrosis), stained for HDAC6 expression; (B) Picrosirius red (PSR) staining of human liver sections in (A) for F0‐F3 classification; (C) HDAC6 staining quantification and statistical analysis between F0–F1 livers and F2–F3 livers (10 patients/group). Scale bar = 100 microns. Data are mean ± SEM, and each datapoint represents a different donor liver. Significance was calculated using an unpaired *t*‐test, and *P < 0.05.

To determine the clinical potential for the HDAC6smi's, we first determined the expression of HDAC6 in archived human liver tissue samples prior to testing the antifibrogenic properties of DR‐3 and FDR2 in a human precision cut liver tissue (hPCLS) model of fibrosis. Human liver tissues obtained from the non‐cancerous margins of resected hepatic secondary colorectal tumors were stained with Picrosirius red (PSR) to enable grading of any incidental fibrosis, this being a not uncommon finding in the absence of a formal diagnosis of a chronic liver disease. Figure 5B shows images of PSR stained liver sections representative of grades F0–F1 (corresponding to either zero to mild fibrosis) and F2–F3 (significant to advanced fibrosis). Figure 5A shows representative staining for HDAC6 in these tissues with evidence of increased expression in association with F2 and F3 grades compared with F0 and F2. Quantification of HDAC6 staining in 20 individual patient samples revealed a statistically significant increase in F2/F3 compared with F0/F1 samples, this confirming a correlative association between the progression of hepatic fibrosis and elevated hepatic expression of HDAC6 (Fig. 5C).

**Fig. 6:**
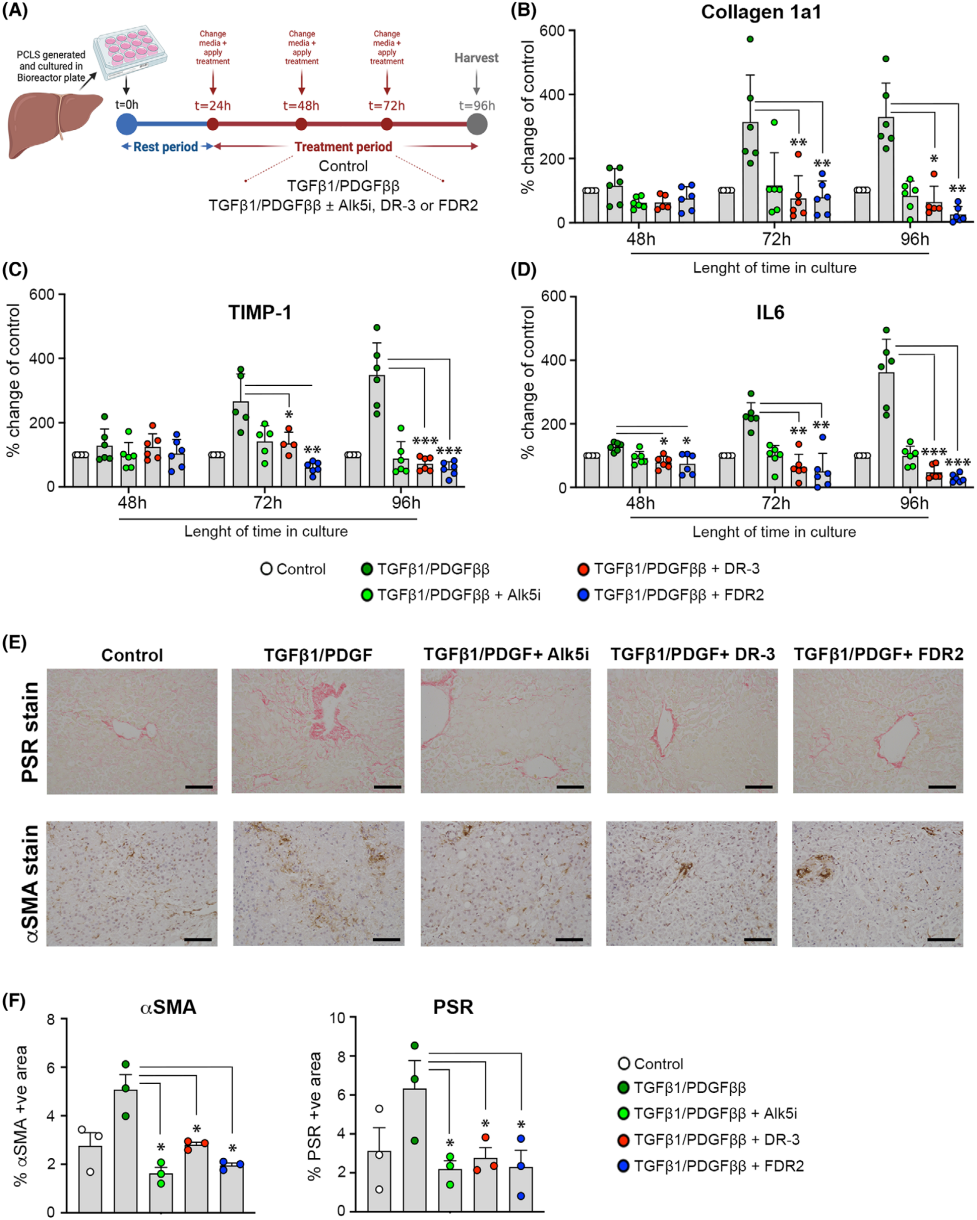
HDAC6smi's exhibit antifibrogenic activities in human precision‐cut liver slice cultures. (A) Schematic representation of the preparation, culture, stimulation, and treatment of human precision cut liver slices (hPCLS). (B–D) Graphs displaying the levels of soluble Col1a1, TIMP‐1, and IL‐6 (expressed as a percentage of control) in the media of bioreactor‐cultured hPCLSs after a 24‐h rest and subsequent 72‐h culture ± fibrogenic stimulation with TGFβ1/PDGFββ ± Alk5i or HDAC6smi DR‐3 and FDR2 (96‐h total culture time). Data are mean ± SEM, from three individual experiments utilizing tissue from *n* = 3 individual donors, performed in duplicate, each datapoint represents a different experimental sample. (E) Representative 200× magnification images of PSR and αSMA stained hPCLSs from three individual donor livers at *t* = 0 and at the end of 96‐h culture as shown in (A) ± TGFβ1/PDGFββ ± Alk5i ± DR‐3/FDR2. Scale bar = 100 microns. (F) Quantification of α‐SMA and PSR positive staining area under different treatment conditions. Statistically significant differences compared to the TGFβ1/PDGFββ group were calculated using a one‐way ANOVA with a Tukey's *post hoc* test and *P < 0.05. Data are mean ± SEM, from *n* = 3 different donor livers. **P < 0.01, ***P < 0.001.

We next determined the antifibrotic potential of DR‐3 and FDR2 in *ex vivo* hPCLS, which are considered the most appropriate human pre‐clinical model for the assessment of the likely clinical benefits of an antifibrotic molecule [43]. In this model, thin (250 μm) slices of precision cut liver tissue obtained from the non‐cancerous margins of hepatic tumor resections are cultured for 96 h in the absence or presence of TGF‐β1 and PDGF, the latter two molecules combining to stimulate a robust fibrogenic reaction in the cultured liver tissue [10]. Under these conditions, and as shown in the schematic in Fig. 6A, the effects of the addition of the HDAC6smi's were compared with the potent antifibrogenic effects of a TGF‐βR/Alk5 inhibitor (Alk5i). All exogenously added factors and drugs were applied after a period of 24 h and were refreshed at 48 and 72 h prior to harvesting at 96 h. Media were sampled from hPCLS at 48, 72, and 96 h to allow dynamic quantification of the soluble fibrogenic proteins Collagen1a1, TIMP‐1, and IL‐6 by ELISA (Fig. 6B–D). As anticipated, all three soluble proteins were detected at increased levels in response to combined TGF‐β1 and PDGF treatment at 72 and 96 h relative to control untreated hPCLS at these timepoints, as well as with hPCLS at the 48 h timepoint, this confirming an expected robust fibrogenic response. As also anticipated, these induced fibrogenic responses were effectively blunted by treatment with 10 μm Alk5i, with in each case levels of soluble proteins being equivalent to those detected in control hPCLS. DR‐3 treatment brought about a similar effect as that seen with Alk5i, such that levels of soluble Collagen1a1, TIMP‐1, and IL‐6 were similar to those seen in control hPCLS. FDR2 also exhibited potent antifibrogenic activity and remarkably suppressed both Collagen1a1 and IL‐6 expression below the basal levels detected in control hPCLS, indicating an ability to suppress background fibrogenesis occurring incidentally in the liver tissues by autocrine mechanisms. Lactate dehydrogenase (LDH) and albumin measurements confirmed that the treatments did not exert toxic effects on the tissue, as evidenced by comparable levels of these markers across all treatment groups; however, albumin levels were slightly lower in TGF‐β1/PDGF‐treated samples toward the end of the culture period, not due to toxicity, but likely due to epithelial‐to‐mesenchymal transition of hepatocytes induced by TGF‐β1/PDGF treatment (Fig. S6A,B). Representative stains for Sirius red and αSMA in hPCLS tissue at the 96 h timepoint confirmed that DR‐3 and FDR2 were able to suppress induced collagen deposition and myofibroblast activation to a similar degree as observed in response to treatment with Alk5i (Fig. 6E). Quantification of stained hPCLS tissues at the 96 h timepoint confirmed the anti‐fibrogenic activities of DR‐3 and FDR2, both compounds exerting similar suppressive effects as were observed in Alk5i‐treated hPCLS (Fig. 6F). Collectively, these data indicate strong potential for DR‐3 and FDR2 in preventing fibrosis progression in the diseased human liver.

## Discussion

Pan‐HDAC inhibitors have restricted therapeutic utility that is limited to cancer due to their broad range of effects. By contrast, small molecule inhibitors that have a combination of good selectivity for HDAC6 over other HDACs and nm potency are anticipated to have wider utility beyond oncology, including tissue fibrosis. In the liver, HDAC6 levels increase with hepatic fibrosis (Fig. 5A), and intriguingly, the staining pattern revealed an increase in HDAC6 in hepatocytes, which suggests a heightened role for HDAC6 in hepatocytes in disease contexts. Notably, HDAC6 staining was minimal in hepatic myofibroblasts/stellate cells, highlighting a cell‐type‐specific expression pattern within the liver. Our observations are consistent with staining data in the Human Protein Atlas, which show HDAC6 staining in hepatocytes within the liver tissue [44, 45] (source: https://www.proteinatlas.org/ENSG00000094631‐HDAC6/tissue/liver#img), and the reported increase in hepatocyte HDAC6 in ischemia/reperfusion injury after liver transplant [46]. However, other research has demonstrated HDAC6's opposing roles in liver regeneration and hepatocarcinogenesis, which underscores its complexity in liver disease [47] and warrants further investigation.

Here we make a case for further investigation of the anti‐fibrotic potential of targeting HDAC6 in the diseased liver by synthesizing two novel small molecule inhibitors and demonstrating their ability to inhibit TGF‐β1 signaling in HSC and suppress induced liver fibrosis in the hPCLS model of human liver fibrosis.

In synthesizing the HDAC6‐specific inhibitors DR‐3 and FDR2, we adhered to a stringent structure–activity relationship paradigm with the objective of achieving selectivity for HDAC6 over HDAC1. The chosen molecular design, particularly the incorporation of aromatic linkers and tailored CAP groups, was predicated on the distinct topology of HDAC6's CD2 active site. This was substantiated by nanomolar IC_50_ values for the two inhibitors, indicating a pronounced affinity for HDAC6 and a 100‐fold selectivity over HDAC1. Although these inhibitory characteristics are comparable with other HDAC6 inhibitors, their selectivity for HDAC6 may be inferior to that of TYA‐018, which is reported to display greater than 2500‐fold selectivity over other zinc‐dependent HDACs [48]. Of note, the latter molecule has shown promise in the context of heart disease and is reported to have anti‐fibrotic properties, confirming the rationale for targeting HDAC6 in chronic disease [35, 48].

As few as 10% of phase 1 drugs receive FDA approval [49]. Animal models are frequently used to test anti‐fibrotic therapies, but these models have limitations; they often fail to faithfully recreate the disease pathology, are performed in young mice, and are limited by a species barrier. Therefore, the community is transitioning towards non‐animal alternative technologies for their pre‐clinical models to provide more accurate and translationally relevant data for evaluating drug safety and efficacy [50]. With regard to *ex vivo* models, hPCLS offer a powerful facsimile in which the cellular complexity of hepatic fibrosis can be modeled in the context of the aged human liver [10, 51, 52]. Adoption of hPCLS has also significantly increased following the FDA report that animal testing is no longer required before human drug trials [53]. Here, both inhibitors demonstrated an ability to suppress fibrogenesis as evidenced by blunting the production of soluble fibrogenic biomarkers in hPCLS media and inhibiting formation of fibrotic matrix in the hepatic parenchyma. Importantly, the hPCLS model is gaining considerable traction as an alternative pre‐clinical model to animal models of liver damage for predicting the efficacy of novel anti‐fibrotic molecules. Hence, the potent effects of DR‐3 and FDR2 in this model are encouraging and warrant future investigations on pharmacological suppression of HDAC6 in CLD.

Important limitations of this study are the lack of examination of the efficacy of DR‐3 and FDR2 in mouse or human models of MASH, which adds the complication of the impacts of steatosis, and an absence of side‐by‐side comparisons of the specificity, potency, and anti‐fibrogenic activities of DR‐3 and FDR2 with other HDAC6 inhibitors such as TYA‐018. In addition, while studies of FDR2 in hPCLS indicated an ability to suppress existing incidental fibrosis, we have not formally tested its ability to reverse fibrosis. Finally, while we have shown that DR‐3 and FDR2 inhibit tubulin acetylation, we have not experimentally confirmed the inhibition of TGF‐β1 target gene expression or carried out more extensive mechanistic investigations that will be required to justify the progression of HDAC6 inhibitors to clinical studies in CLD.

In summary, HDAC6 is expressed at elevated levels in CLD, and on the basis of these studies using two novel selective small molecule inhibitors, it is worthy of further mechanistic and pharmacological investigation as a profibrogenic protein deacetylase in the context of liver damage but also in other fibrosing conditions.

## Materials and methods

For detailed methods outlining the chemical synthesis of novel compounds DR‐3 and FDR2, and the NMR spectra (Figs S1–S5), see Appendix S1.

### HDAC1 and HDAC6 enzymatic activity assay

Deacetylase activity of compounds DR2 and FDR2 was measured using the HDAC‐Glo I/II assay kit (G6420) (Promega, Madison, WI, USA). Briefly, compounds DR‐3 and FDR2 were serially diluted in HDAC‐Glo™ I/II Buffer at appropriate concentrations (final volume 25 μL), then 25 μL of 0.2 nm HDAC enzyme (human recombinant C‐ter‐GST‐HDAC 1 (H83‐30G)) and 0.2 nm N‐ter‐GST‐HDAC 6 (50006) (BPS Bioscience, San Diego, CA, USA) was added. The HDAC1 and HDAC6 deacetylase activity assays were then performed following the manufacturer's protocol and the luminescent values of the deacetylase activity were measured using a Filtermax F5 multi‐mode plate reader.

### Cell culture

Primary rat HSCs were isolated from the liver of normal adult male Sprague–Dawley rats as previously described [54]. Briefly, livers were perfused sequentially with pronase and collagenase, then tissue was passed through a Nybolt filter to obtain a single‐cell suspension, and buoyant HSCs were isolated by discontinuous density centrifugation in 11.5% Optiprep (Life Technologies, Paisley, UK). Rat HSCs were cultured on 10 cm dishes in Dulbecco's modified Eagle's medium (DMEM; Life Technologies) supplemented with 16% fetal bovine serum, 1 mm pyruvate, 2 mm glutamine, 100 U·mL^−1^ penicillin, and 100 μg·mL^−1^ streptomycin (Life Technologies). LX‐2 cells were a gift of Prof Scott Freidman (Mount Sinai, USA). LX‐2 cells were cultured as above, apart from FBS, which was supplemented at 2%. All cells were maintained in an incubator at 37 °C with 5% CO_2_.

### Ethics

Human liver tissue was obtained under full ethical approval from the Newcastle and North Tyneside Regional ethics committee (H10/H0906/41) and through the CEPA biobank (17/NE/0070) and used subject to patients written and informed consent. The study methodologies conform to the standards set by the Declaration of Helsinki. Control human liver tissue for PCLS was collected from patients undergoing cancer surgical resections and surplus to pathology requirements. Animal work was approved by the Newcastle Ethical Review Committee and performed under a UK Home Office license (P019959F0) in accordance with the ARRIVE guidelines. Adult male Sprague–Dawley rats were purchased from Charles River, UK, and housed in pathogen‐free conditions on a 12 h light/dark cycle with free access to food and water.

### Precision cut liver slices

Human Precision cut liver slices (PCLS) were generated and cultured as previously described [10]. After a 24 h rest period, human PCLS were stimulated with 3 ng·mL^−1^ of transforming growth factor beta 1 (TGFβ1) and 50 ng·mL^−1^ of platelet‐derived growth factor (PDGFββ; PeproTech, London, UK) for a total of 72 h to induce fibrogenesis. All PCLS were cultured at 37 °C, supplemented with 5% CO_2_ and media ± stimulation ± therapy. During the 72 h, the media were replenished daily, and conditioned media from the previous 24 h period were collected and stored for measurements of soluble fibrogenic and inflammatory factors. TGFβ1/PDGFββ stimulated hPCLS were treated ± therapies: 10 μm SB‐525334 (an activin receptor‐like kinase 5 (Alk5) inhibitor (Alk5i)), 100 nm FDR2, or 100 nm DR‐3.

### Immunocytochemistry

LX‐2 were grown on sterile glass coverslips in 12‐well plates treated with 100 nm of DR‐3 or FDR2 compound, 24 h before 15‐ or 30‐min stimulation with 5 ng·mL^−1^ of TGFβ1. Cells were fixed, washed 3× with PBS, then blocked with Fetal bovine serum for 1 h. Cells were then stained with Phospho‐SMAD3 (Ser423/425) (C25A9) (mAb #9520; Cell Signalling, Leiden, The Netherlands), at a 1 : 500 dilution, for 2 h at RT, and then washed 3× with PBS. Next, Goat anti‐rabbit Alexafluor 488 secondary antibody (A11008; Invitrogen, Paisley, UK) was diluted in 1%FBS (1 : 2000) and incubated with cells for 2 h. Cells were next washed 3× with PBS and incubated for 10 min with DAPI in PBS at RT. After 3× washes in ddH_2_O, coverslips were mounted onto glass slides using Prolong Gold Antifade medium.

LX‐2 cells were grown on sterile glass coverslips in 12‐well plates treated with 100 nm of DR‐3 or FDR2 compound for 24 or 48 h. After that time, cells were fixed and blocked as above. After blocking in 1% FBS, fixed cells were incubated with anti‐rabbit Acetylated tubulin mAb (5335S; Cell Signalling) 1 : 500 in 1% FBS for 3 h at RT or FITC‐conjugated αSMA mouse mAb 1 : 1000 (F3777; Sigma, Gillingham, UK). After that time, cells were washed 3× with PBS and incubated with secondary antibody anti‐rabbit Alexa Fluor 594 secondary antibody 1 : 2000 (594 A11037) for 2 h at RT. Cells were next washed 3× with PBS and incubated for 10 min with DAPI in PBS at RT. After 3× washes in ddH2O, coverslips were mounted onto glass slides immediately using Prolong Gold Antifade mounting medium.

### Immunofluorescent imaging and analysis

Images of p‐Smad3 IF stained LX‐2 cells were taken on a Zeiss LSM800 confocal (Zeiss, Cambridge, UK) using an EC Plan Neofluar 40× 1.3NA oil immersion lens with a fixed pinhole of 100 μm (15.2 μm optical section for DAPI and 8.3 μm optical section for Alexafluor 488/ FITC respectively) using zen blue v3.8 software (Zeiss, Cambridge, UK). Single plane images were collected at Nyquist sampling (76 nm pixels) with 4× averaging using 405 and 488 nm solid state lasers sequentially with 410–470 nm and 505–585 nm detection windows selected for DAPI and Alexafluor 488 respectively. Ten random images were taken of each coverslip and analyzed using the Zen image analysis Zone of Influence (ZOI) tool by segmenting automatically using Otsu or Triangle thresholding of the DAPI channel to detect the nuclei and a 30‐pixel radius ZOI outside the nuclei to represent the cytoplasm. Mean intensities for the Alexafluor 488 channel were taken for both nuclear and their respective cytoplasmic regions of interest. Data analysis was carried out by using the FlowJo LLC software (Ashland, OR, USA). The intensity of the fluorescent stain in single cells was quantified by imagej (National Institute Of Health, Bethesda, MD, USA). For Fig. 4A, the image look‐up tables for the nuclear counterstain channel for the TGFβ + DR3 (individual image and montage) were adjusted to ensure the contrast of the images presented were visible in the final figures. No impact was made to the analyzed channels or to the final results obtained.

Images of acetylated tubulin‐stained LX2‐stained cells were taken on a Zeiss LSM800 confocal using an EC Plan Neofluar 40× 1.3NA oil immersion lens with a fixed pinhole of 100 μm (15.2 μm optical section for DAPI and 8.3 μm optical section for Alexafluor 594 respectively).

### Histology, immunohistochemistry and image analysis

Staining was performed on formalin‐fixed, paraffin‐embedded 5 μm thick sections from human liver or hPCLS. Slides were stained with 0.1% Picrosirius Red using established protocols. For immunohistochemistry, slides were deparaffinized, then endogenous peroxidase activity was blocked using a 0.6% hydrogen peroxide/methanol solution. Heat‐mediated antigen retrieval was performed using antigen unmasking solution (Vector Laboratories, Newark, CA, USA). Slides were mounted into sequenzas, then endogenous avidin and biotin were blocked for 20 min using an Avidin/Biotin Blocking Kit (Vector Laboratories). Non‐specific binding was blocked using 20% swine serum for 30 min, and then the primary antibody, FITC conjugated αSMA 1 : 1000 (F3777; Sigma) or HDAC6 1 : 500 (clone D2E5) (Rabbit mAb #7558; Cell Signalling), was added and incubated overnight at 4 °C. The next day, slides were PBS washed and incubated with biotinylated goat anti‐fluorescein 1 : 300 (BA‐0601 Vector) or biotinylated swine anti‐rabbit 1 : 200 (eo353; Dako (Agilent technologies), Cheshire, UK) and incubated at RT for 2 h. Slides were then PBS washed and incubated with Vectastain Elite ABC Reagent, before visualizing the stain using a DAB peroxidase substrate kit and counterstaining with Mayers hematoxylin and pertex mounting.

Stained liver sections were imaged at 100× or 200× using a Nikon Eclipse Upright microscope, and thresholding of HDAC6 stain was performed using NIS‐Elements BR analysis software. A minimum of 10 non‐overlapping fields were analyzed per slide.

### RNA isolation, cDNA synthesis and RT–PCR

RNA was extracted from rat HSC treated ± DR‐3 or FDR2, using the QIAGEN RNeasy Mini kit (QIAGEN, Manchester, UK) according to the manufacturer's instructions. cDNA synthesis and quantitative SYBR Green real‐time PCR were performed as previously described [55]. The primers are listed in Table S1.

### Statistical analysis

All data are presented as means ± SEM, and graphpad prism version 10 (Boston, MA, USA) was used to perform an unpaired *t*‐test or analysis of variance with a Tukey's *post hoc* test for unmatched samples, or a One‐way analysis of variance with a Tukey's *post hoc* test. *P < 0.05, **P < 0.01, ***P < 0.001, or ****P < 0.0001 were considered statistically significant.

## Author contributions

MTB and DR, contributed to the conceptualization of the project, performed experiments, and analyzed data. MP, KN, BR, and DR, designed and synthesized the HDAC6smi compounds. HP, EG, ALC, RS, SH, and LAB, performed experiments, analyzed data. GN and DB performed imaging and analyzed data. SMR, JF, JM, SAW, CW, SP, JH, RT, WA, and RF provided human tissue samples. FO, DR, DAM, and J Mann, contributed to the conceptualization of the project, performed experiments, analyzed data, created the figures, and wrote the manuscript. All authors contributed to the drafting and editing of the manuscript.

## Conflict of interest

FO, DAM, LAB, and JM are founders, shareholders, and directors of FibroFind Ltd.

## Supporting information


**Appendix S1.** Supplementary materials and methods.
**Fig. S1.** ^1^HNMR spectrum of the compound DR‐3.
**Fig. S2.** ^13^C NMR spectrum of the compound DR‐3.
**Fig. S3.** ^1^HNMR spectrum of the compound FDR2.
**Fig. S4.** ^13^CNMR spectrum of the compound FDR2.
**Fig. S5.** DEPT spectrum of the compound FDR2.
**Fig. S6.** Viability and function of human precision‐cut liver slice cultures are not altered HDAC6smi's.
**Table S1.** Primers sequences used in this study.

## Data Availability

Data related to the synthesis of the HDAC6 small molecule inhibitors (DR‐3 and FDR2) and structural characterization are available within the article and the Supporting Information (NMR characterization – Figs S1–S5).
