# Supplementary material for: Pharmacological manipulation of liver fibrosis progression using novel HDAC6 inhibitors
Source: FEBS J. 2025 Mar 14;292(13):3397–411. doi: 10.1111/febs.70062 (PMC12220842; doi:10.1111/febs.70062)
Supplement: Supplementary file 1 — Appendix S1. Supplementary materials and methods. Fig. S1. 1HNMR spectrum of the compound DR‐3. Fig. S2. 13C NMR spectrum of the compound DR‐3. Fig. S3. 1HNMR spectrum of the compound FDR2. Fig. S4. 13CNMR spectrum of the compound FDR2. Fig. S5. DEPT spectrum of the compound FDR2. Fig. S6. Viability and function of human precision‐cut liver slice cultures are not altered HDAC6smi's. Table S1. Primers sequences used in this study. [file FEBS-292-3397-s001.docx]

**Pharmacological manipulation of liver fibrosis progression using novel HDAC6 inhibitors**

**Supplementary materials and methods**

**Structure-based modelling - HDAC6 inhibitors**

The synthesized HDAC6 inhibitors DR-3 and FDR2 were sketched in ChemDraw software v. 7.0.1. The protonation states (pH=7.4) were calculated in MarvinSketch 6.1.0 (<https://chemaxon.com/>). To obtain the three-dimensional conformation of the studied HDAC inhibitors, energy minimization was performed in the gas phase using Chem3D Ultra 7.0 software (utilizing the Hartree-Fock 3-21G method via Gaussian 7.0.0). Deprotonated hydroxamic acids from the optimized 3D structures were used for molecular docking analysis. Additionally, the crystal structure of the second catalytic domain of human histone deacetylase 6 (PDB: 5EDU) was downloaded from the PDB website.

The X-ray crystal structures of HDAC1 and HDAC6 enzymes were protonated under physiological conditions using Play-Molecule’s protein prepare wizard (<https://www.playmolecule.com/>). Catalytic water molecule in HDAC6 was retained for docking calculations. The three-dimensional structures of the inhibitors were treated flexible during docking where the structure of the HDAC6 was kept rigid. Docking procedure was carried in metalloenzyme configuration mode in GOLD software v.5.8.1 with *ChemScore* calculated as scoring function. The validity of the docking procedures was examined by inspecting the Root-Mean-Square Deviation value (RMSD <2Å) and docking results were visualized in Pymol molecular visualization software (www.pymol.org).

**Synthesis of Novel HDAC6 inhibitors DR-3 & FDR2 – general methods**

Solvents and chemicals were purchased from commercial vendors and used without further purification. ^1^H and ^13^C nuclear magnetic resonance (NMR) spectra were recorded at room temperature on a Bruker Ascend 400 (400 MHz) spectrometer, using TMS as an internal standard. Chemical shifts are reported in ppm (δ). Spin multiplicities are described as s (singlet), d (doublet), dd (double doublet), t (triplet), or m (multiplet). Coupling constants are reported in hertz (Hz). Low-resolution mass spectra were obtained using TSQ Quantum Access MAX triple quadrupole mass analyser (Thermo Fisher Scientific Inc., San Jose, CA, USA) with heated electron spray ionization source (HESI).

**Synthesis of compound DR-3 (scheme shown in Figure 1B)**

**Synthesis of 4-morpholino-4-ylmethyl-benzoic acid methyl ester (compound 2)**

In a round bottom flask 4-bromomethylbenzoic acid methyl ester, **1** (230 mg; 1.0040 mmol), K_2_CO_3_ (235.9 mg; 1.7069 mmol) and morpholine (96.0 mg, 1.1045mmol) were dissolved in dry acetonitrile (10 mL) and mixed at room temperature for 18 hours. After this time, the mixture was filtered through Celite pad and washed with ethyl acetate. The compound **2** was isolated without further purification as a white solid (233 mg; 0.9903 mmol; yield 98.6%): **^1^H NMR** (400 MHz, CDCl_3_) δ 7.96 (d, J = 7.5 Hz, 2H), 7.38 (d, J = 7.9 Hz, 2H), 3.87 (s, 3H), 3.73 – 3.64 (m, 4H), 3.51 (s, 2H), 2.47 – 2.36 (m, 4H);

**^13^C NMR** (101 MHz, CDCl_3_) δ 167.0, 143.4, 129.6, 129.1, 128.9, 67.0, 63.0, 53.7, 52.1.

**Synthesis of N-Hydroxy-4-morpholin-4-ylmethyl-benzamide (compound DR-3)**

Solutions of hydroxylamine hydrochloride (64.97 mg; 0.9351 mmol) in 5 mL of MeOH, and of KOH (104.93 mg; 1.8701 mmol) in 5 mL of MeOH, were prepared. Both were cooled on ice bath, and the KOH solution was added with shaking to the hydroxylamine hydrochloride solution. The mixture was allowed to stand in an ice bath for 30 minutes to ensure complete precipitation of potassium chloride. The mixture was filtered and the filtrate was added to the compound 2 (100 mg, 0.4250 mmol) in 25 mL flask. Additional potassium hydroxide was added to ensure a basic solution (pH~10). After 12 hours with stirring at room temperature, MeOH was evaporated *in vacuo* and 20 mL of water was added to the residue, which was neutralized with 2 M HCl and extracted with EtOAc (3 x 10 mL). The organic layers were separated, washed with brine, dried over anhydrous Na_2_SO_4_, concentrated under vacuum. The crude product was purified by flash chromatography (0 – 10% MeOH in DCM) to afford desired product as a pale yellow solid (60 mg; 0.2539 mmol; yield 59.7%): **^1^H NMR (400 MHz, DMSO) δ** 11.16 (s, 1H), 9.00 (s, 1H), 7.80 – 7.60 (m, 2H), 7.52 – 7.26 (m, 2H), 3.57 (s, 4H), 3.50 (s, 2H), 2.35 (s, 4H); **^13^C NMR (101 MHz, DMSO) δ** 164.6, 141.7, 132.0, 129.2, 127.3, 66.7, 62.4, 53.6.

**MS:** (ESI) calcd for C_12_H_16_N_2_O_3_ 236.2; measured [m/z, M+H]: 237.1

**Synthesis of compound FDR2 (scheme shown in Figure 1C)**

**5,5-diphenyl-imidazolidine-2,4-dione (compound 4).**

Benzil (1 g; 4.7 mmol), urea (0.5 g, 8.3 mmol) and 2.5 mL of KOH solution (65 % w/w) were mixed in 20 mL of ethanol and the reaction was refluxed for 2 hours. After 2h, the reaction mixture was poured into ice water (20 mL), and the precipitate was filtered. Then 6M solution of hydrochloric acid was added to the filtrate until a precipitate was formed. The formed precipitate was filtered, washed with water and dried at room temperature. Compound 4 was obtained as white crystalline solid, 1.01 g, yield 83%: **^1^H NMR** (400 MHz, DMSO): δ 11.12 (s, 1H), 9.34 (s, 1H), 7.37 - 7.45 (m, 10H); **^13^C NMR** (101 MHz, CDCl_3_): δ 174.5, 155.9, 139.7, 128.2, 127.6, 126.4, 70.0

**4-(2,5-Dioxo-4,4-diphenyl-imidazolidin-1-ylmethyl)-benzoic acid methyl ester (compound 5).**

Compound **4** (126.1 mg; 0.5 mmol) was mixed with methyl 4-(bromomethyl)benzoate (126 mg; 0.55 mmol) and K_2_CO_3_ (87 mg; 0.63 mmol) in 5 mL of dry acetone and refluxed overnight. After 18h, acetone was evaporated and the reaction products were dissolved in water, extracted with dichlormethane and purified with flash chromatography (PE : Et_2_O = 1:1). After chromatography, fractions containing the product were evaporated and 162.1 mg of compound **5** was isolated as a white crystalline solid, yield 81% : **^1^H NMR** (400 MHz, CDCl_3_): δ 7.96 (d, J = 6.9 Hz, 2H), 7.39 (d, J = 6,9 Hz, 2H), 7.32 (s, 10H), 4.75 (s, 2H), 3.90 (s, 3H); **^13^C NMR** (101 MHz, CDCl_3_): δ 172.8, 166.4, 155.7, 139.9, 138.9, 129.7, 129.2, 128.1, 127.5, 127.3, 126.3, 70.2, 52.1, 41.8.

**4-(2,5-Dioxo-4,4-diphenyl-imidazolidin-1-ylmethyl)-benzoic acid (compound 6).**

Compound **5** (75.0 mg; 0.1873 mmol) was mixed with LiOH (13,1 mg; 0.5431 mmol) in a round bottom flask and dissolved in a mixture of tetrahydrofuran/water = 1:1 (v/v; 3 mL). The reaction was stirred overnight at room temperature. After 18h, the tetrahydrofuran was evaporated and *pH* of the remaining water solution was adjusted to pH=3. Compound 6 was extracted with ethyl-acetate, washed with brine (3×5 mL) and dried over anhydrous Na_2_SO_4_. The organic solvent was removed under reduced pressure. Compound 6 (73 mg) was isolated as a white crystalline compound in quantitative yield (~100%): **^1^H NMR (400 MHz, DMSO)** δ 12.91 (s, 1H), 9.75 (s, 1H), 7.86 (d, J = 8.1 Hz, 2H), 7.43 – 7.25 (m, 12H), 4.68 (s, 2H); **^13^C NMR** (101 MHz, DMSO) δ 172.8, 166.6, 154.4, 141.2, 138.9, 130.5, 129.6, 129.3, 128.4, 128.0, 127.1, 126.3, 69.1.

**4-(2,5-Dioxo-4,4-diphenyl-imidazolidin-1-ylmethyl)-N-(tetrahydro-pyran-2-yloxy)-benzamide, (compound 7)**

Compound 6 (50 mg; 0.1294 mmol) was disolved in CH_2_Cl_2_ (3 mL) and EDCI•HCl (35 mg, 0.1811 mmol), anhydrous HOBt (31.5 mg; 0.2329 mmol), Et_3_N (24 mg; 0.2330 mmol), *O*-(Tetrahydro-2H-pyran-2-yl) hydroxylamine (23 mg; 0.1941 mmol) were added. The mixture was stirred at 40 ºC overnight. After that time, the reaction mixture was transferred to a separatory funnel, washed with brine (3 x 5 mL), dried over anhydrous Na_2_SO_4_ and concentrated *in vacuo*. The compound 7 was purified with flash chromatography (1:1 EtOAc/hexane with 10% methanol) to afford white solid (37.8 mg; yield 62.8%): **^1^H NMR** (400 MHz, DMSO) δ 11.59 (s, 1H), 9.79 (s, 1H), 7.70 (d, J = 8.0 Hz, 2H), 7.33-7.43 (m, 10H), 7.29 (d, J = 8.0 Hz, 2H), 4.97 (s, 1H), 4.69 (s, 2H), 3.51 (d, J = 10.8 Hz, 1H), 1.71 (s, 3H), 1.53 (s, 4H); **^13^C NMR** (101 MHz, DMSO) δ 173.5, 164.4, 155.5, 140.4, 139.9, 132.1, 129.1, 128.7, 128.0, 127.6, 127.0, 101.4, 69.7, 61.8, 41.7, 28.3, 25.2, 18.7.

***4-(2,5-Dioxo-4,4-diphenyl-imidazolidin-1-ylmethyl)-N-hydroxy-benzamide*, (compound FDR2)**

Compound 7 (30 mg; 0.0644 mmol) and p-toluenesulfonic acid (3.8 mg; 0.0199 mmol) were dissolved in 2-propanol (2 mL). The reaction mixture was stirred overnight at room temperature. Solvent was evaporated *in vacuo* and compound FDR2 was purified by flash chromatography (hexane /EtOAC = 2/1 and DCM/MeOH 95/5, v/v) to afford pale yellow solid (11.1 mg; yield 43%): **^1^H NMR** (400 MHz, MeOD) δ 7.68 (d, *J* = 8.1 Hz, 2H), 7.45 – 7.27 (m, 12H), 4.76 (s, 2H); **^13^C NMR** (101 MHz, MeOD) δ 175.2, 167.8, 157.6, 141.5, 140.8, 133.1, 129.7, 129.5, 129.1, 128.5, 128.0, 71.5, 42.7.

MS-ESI (m/z) calculated for C_23_H_19_N_3_O_4_ [M+H]^+^ 402.4, found: 402.1

***Albumin ELISA and LDH assay***

Quantification of human albumin or lactate dehydrogenase (LDH) in the human PCLS culture media was performed using either the Human Albumin DuoSet ELISA kit (R&D Systems, DY1455) or CyQUANT LDH assay (Invitrogen), according to the manufacturer’s instructions.

**Figure S1.** ^1^HNMR spectrum of the compound DR-3


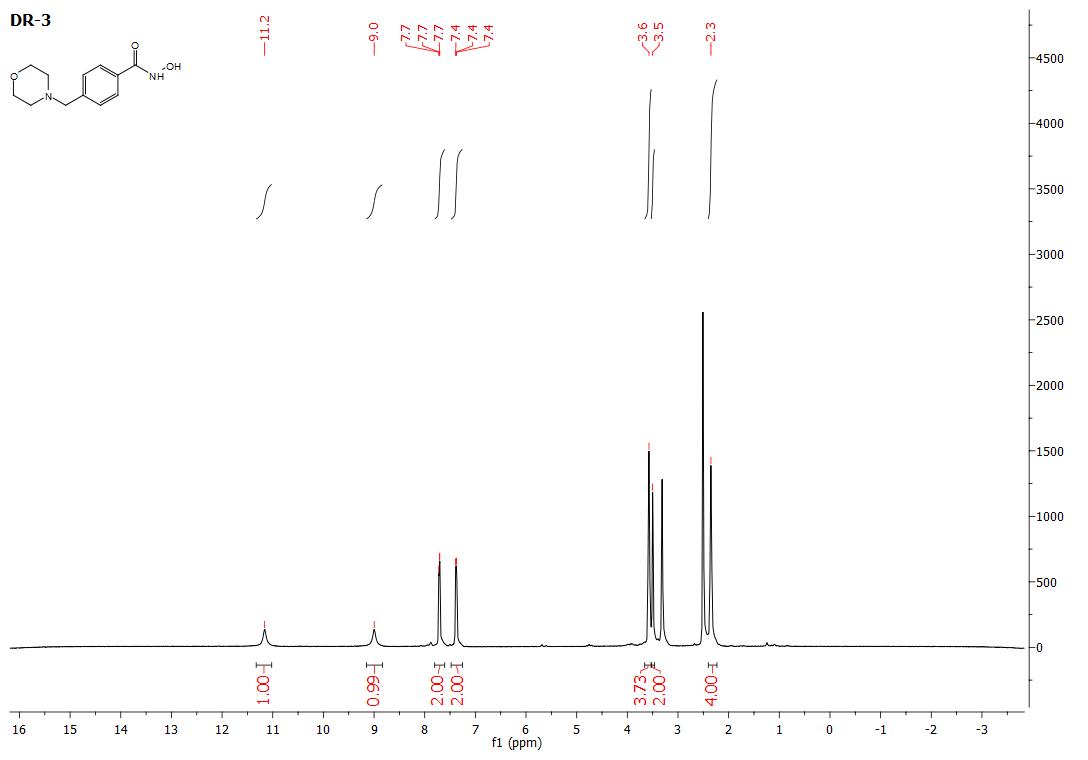


**Figure S2.** ^13^C NMR spectrum of the compound DR-3


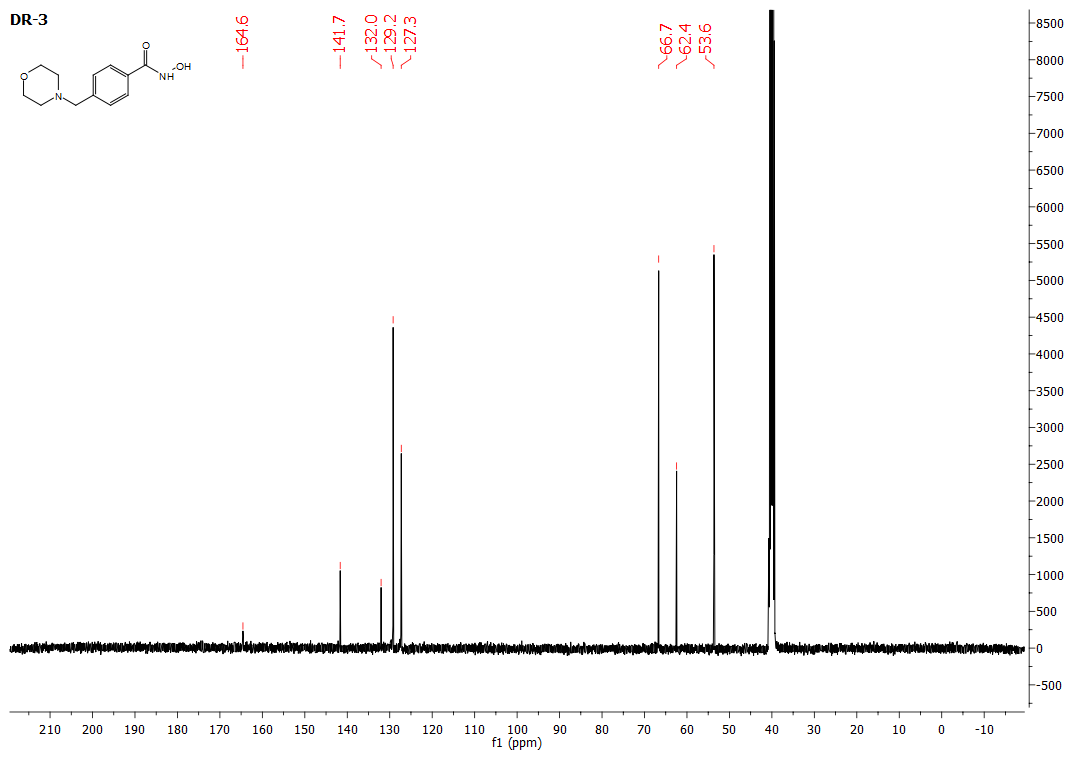


**Figure S3.** ^1^HNMR spectrum of the compound FDR2


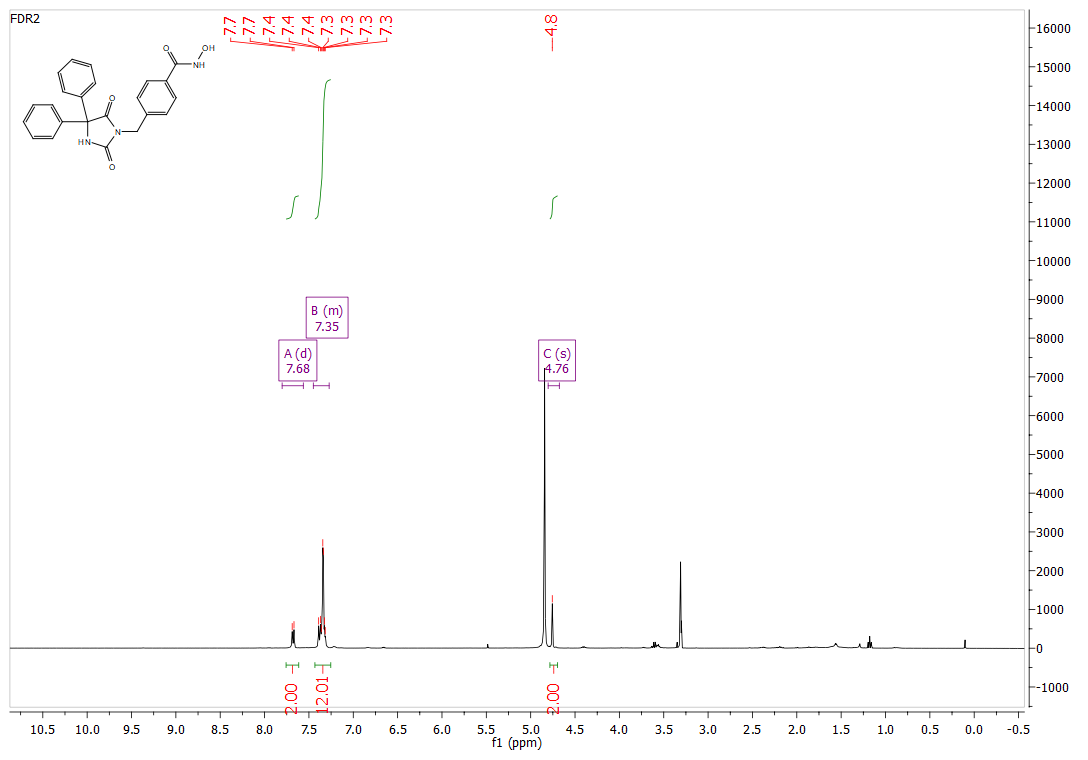


**Figure S4.** ^13^CNMR spectrum of the compound FDR2


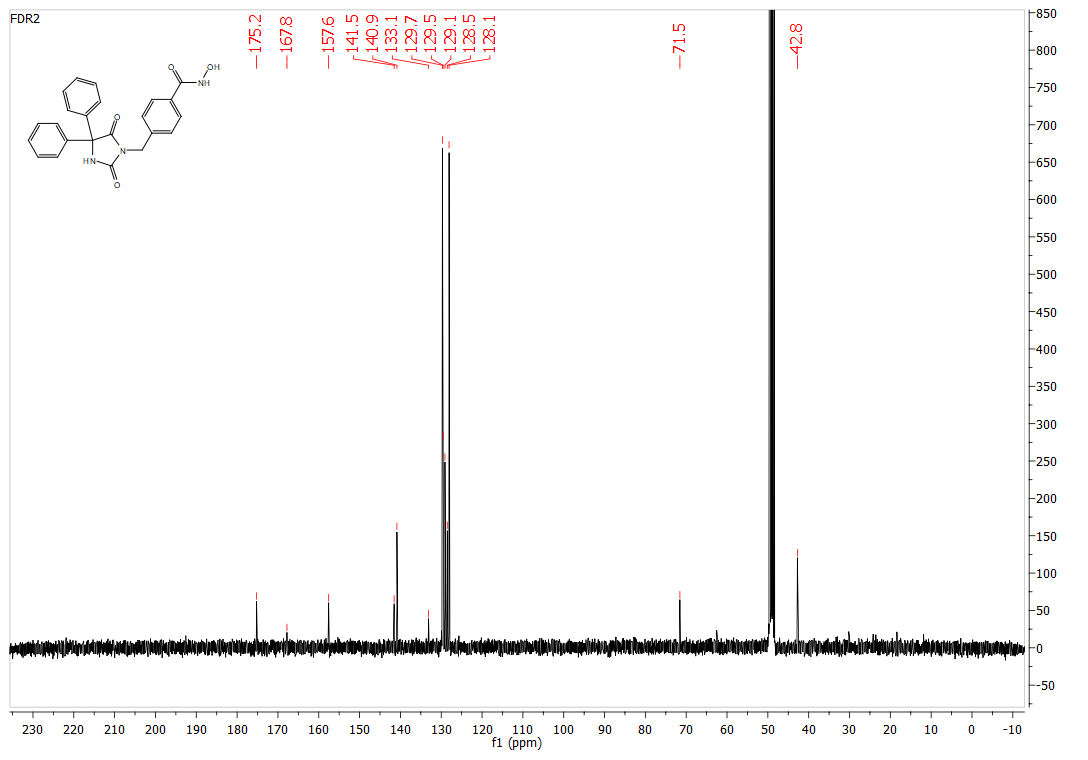


**Figure S5.** DEPT spectrum of the compound FDR2


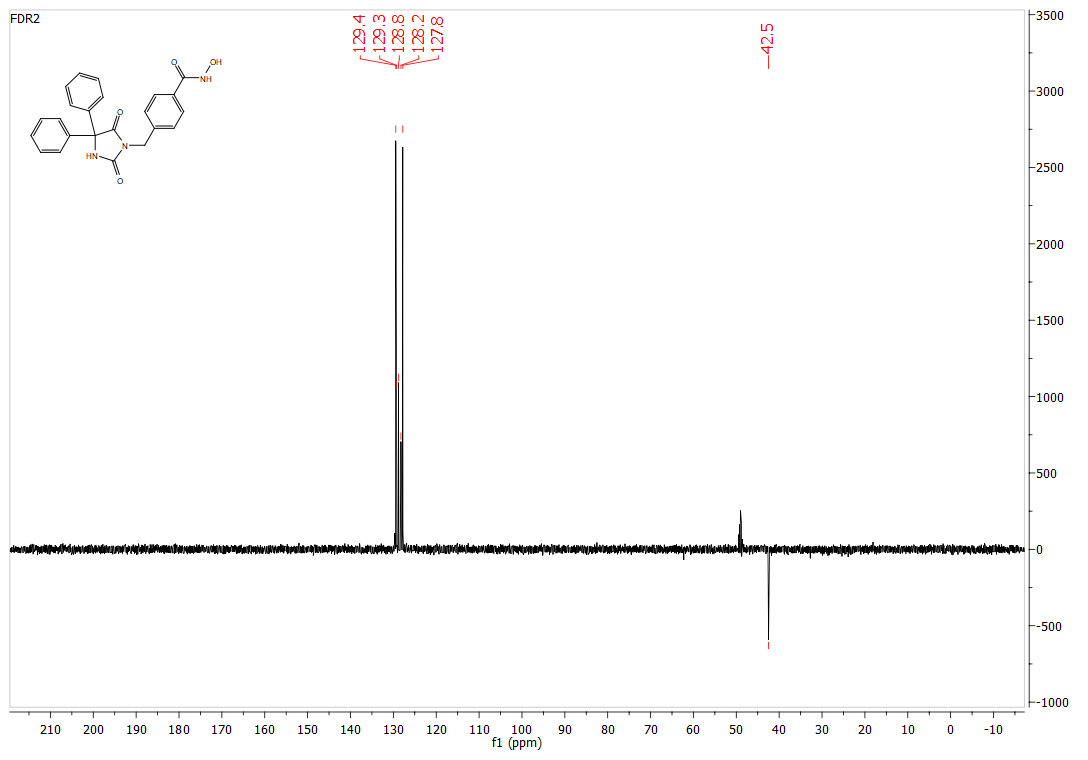


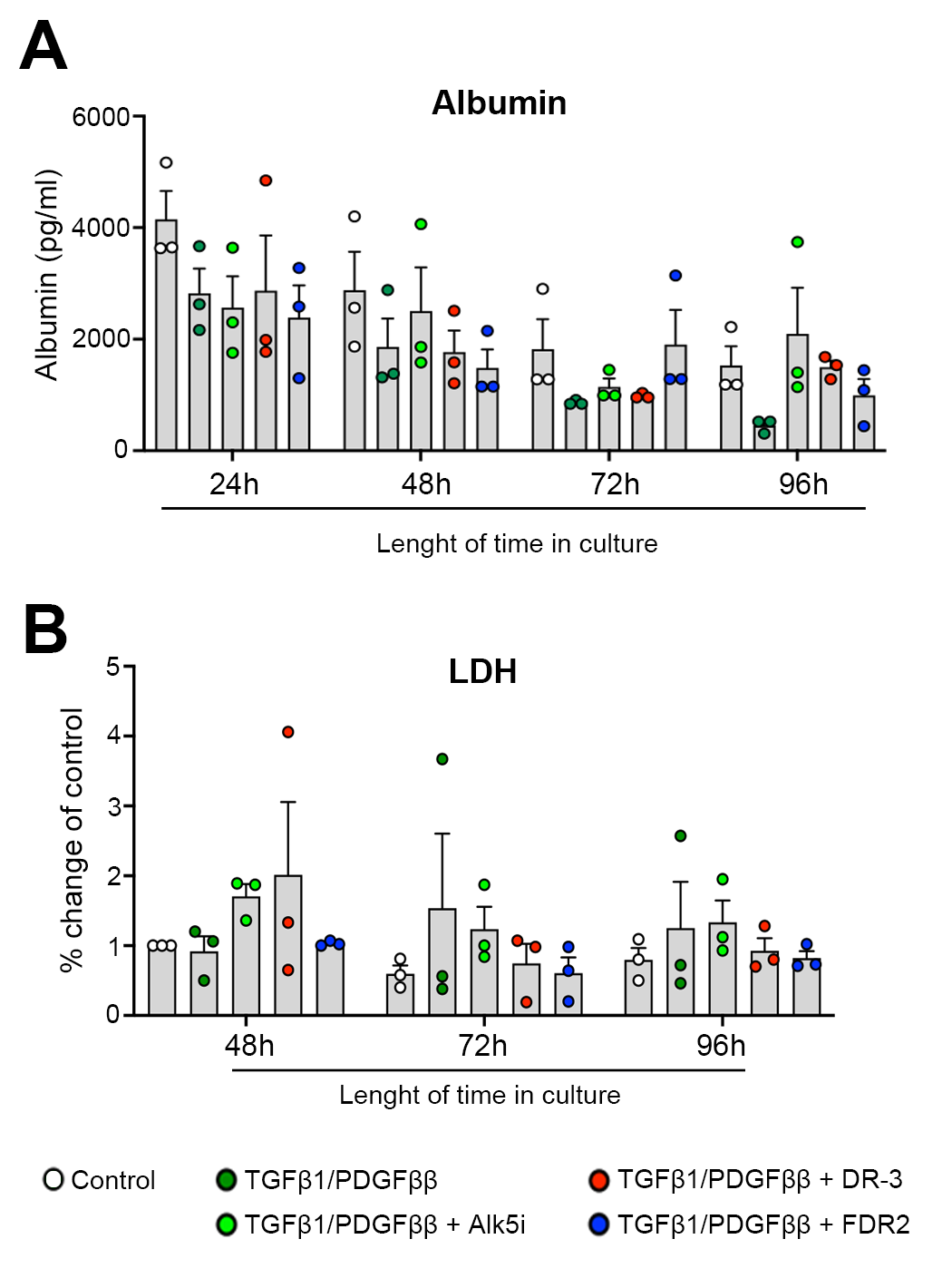


**Figure S6. Viability and Function of Human Precision-Cut Liver Slice Cultures are not altered HDAC6smi’s.**

A) Graph shows secreted albumin (pg/mL) levels in media of bioreactor-cultured hPCLSs during a 24-hour (rest condition) and then subsequent 72-hour culture ± fibrogenic stimulation with TGFβ1/PDGFββ ± Alk5i or HDAC6smi DR-3 and FDR2 (96-hour total culture time). B) Graph shows LDH levels (expressed as a percentage of control) in the media of bioreactor-cultured hPCLSs after a 24-hour rest period and subsequent 72-hour culture ± fibrogenic stimulation with TGFβ1/PDGFββ ± Alk5i or HDAC6smi DR-3 and FDR2 (96-hour total culture time). Data are mean ± s.e.m, from n =3 different donor livers.

| Gene | *Forward primer (5’🡪3’)* | *Reverse primer (5’🡪3’)* |
| --- | --- | --- |
| Human | | |
| GADPH | ATGGGGAAGGTGAAGGTC | GGGGTCATTGATGGCAACAATA |
| COL1A1 | TGAGCCAGCAGATCGAGA | ACCAGTCTCCATGTTGCAGA |
| α-SMA | ACTGAGCGTGGCTATTCCTTCGTT | GCAGTGGCCATCTCATTTTCA |
| TIMP-1 | ACTTCCACAGGTCCCACAAC | CATTCCTCACAGCCAACAGT |
| Rat | | |
| Gadph | GCAAGAGAGAGGCCCTCAG | TGTGAGGGAGATGCTCAGTG |
| Col1a1 | TTCACCTACAGCACGCTTGTG | GATGACTGTCTTGCCCCAAGTT |
| α-Sma | CGAAGCGCAGAGCAAGAGA | CATGTCGTCCCAGTTGGTGAT |
| Timp-1 | TCTGGCATCCTCTTGGTTGCT | TAACCAGGTCCGAGTTGCAG |

**Supplementary table 1:** Primers sequences used in this study.
